# Auditory Steady State Response; nature and utility as a translational science tool

**DOI:** 10.1038/s41598-019-44936-3

**Published:** 2019-06-11

**Authors:** Naoki Kozono, Sokichi Honda, Mariko Tada, Kenji Kirihara, Zhilei Zhao, Seiichiro Jinde, Takanori Uka, Hiroshi Yamada, Mitsuyuki Matsumoto, Kiyoto Kasai, Takuma Mihara

**Affiliations:** 1 Candidate Discovery Science Labs., Drug Discovery Research, Astellas Pharma Inc., 21 Miyukigaoka, Tsukuba-shi, Ibaraki, 305-8585 Japan; 2 Department of Neuropsychiatry, Graduate School of Medicine, The University of Tokyo, 7-3-1 Hongo, Bunkyo-ku, Tokyo, 113-8655 Japan; 3 International Research Center for Neurointelligence (WPI-IRCN), UTIAS, The University of Tokyo, 7-3-1 Hongo, Bunkyo-ku, Tokyo, 113-0033 Japan; 4 Department of Integrative Physiology, Graduate School of Medicine, University of Yamanashi, 1110 Shimokato, Chuo, Yamanashi, 409-3898 Japan

**Keywords:** Predictive markers, Schizophrenia, Sensory processing

## Abstract

The auditory steady-state response (ASSR) has been used to detect auditory processing deficits in patients with psychiatric disorders. However, the methodology of ASSR recording from the brain surface has not been standardized in preclinical studies, limiting its use as a translational biomarker. The sites of maximal ASSR in humans are the vertex and/or middle frontal area, although it has been suggested that the auditory cortex is the source of the ASSR. We constructed and validated novel methods for ASSR recording using a switchable pedestal which allows ASSR recording alternatively from temporal or parietal cortex with a wide range of frequencies in freely moving rats. We further evaluated ASSR as a translational tool by assessing the effect of ketamine. The ASSR measured at parietal cortex did not show clear event-related spectral perturbation (ERSP) or inter-trial coherence (ITC) in any frequency bands or a change with ketamine. In contrast, the ASSR at temporal cortex showed clear ERSP and ITC where 40 Hz was maximal in both gamma-band frequencies. Ketamine exerted a biphasic effect in ERSP at gamma bands. These findings suggest that temporal cortex recording with a wide frequency range is a robust methodology to detect ASSR, potentially enabling application as a translational biomarker in psychiatric and developmental disorders.

## Introduction

Electroencephalographically (EEG)-detectable event-related potential (ERP) analysis can reveal sensory processing deficits in patients with psychiatric disorders^1^. The auditory steady-state response (ASSR) has been used to study auditory temporal processing, hearing screening^2^, and to monitor the state of arousal during anesthesia^3^. Recently, ASSR has been proposed as a biomarker for psychiatric disorders such as schizophrenia^1^ and developmental disorders^4^.

**Figure 1:**
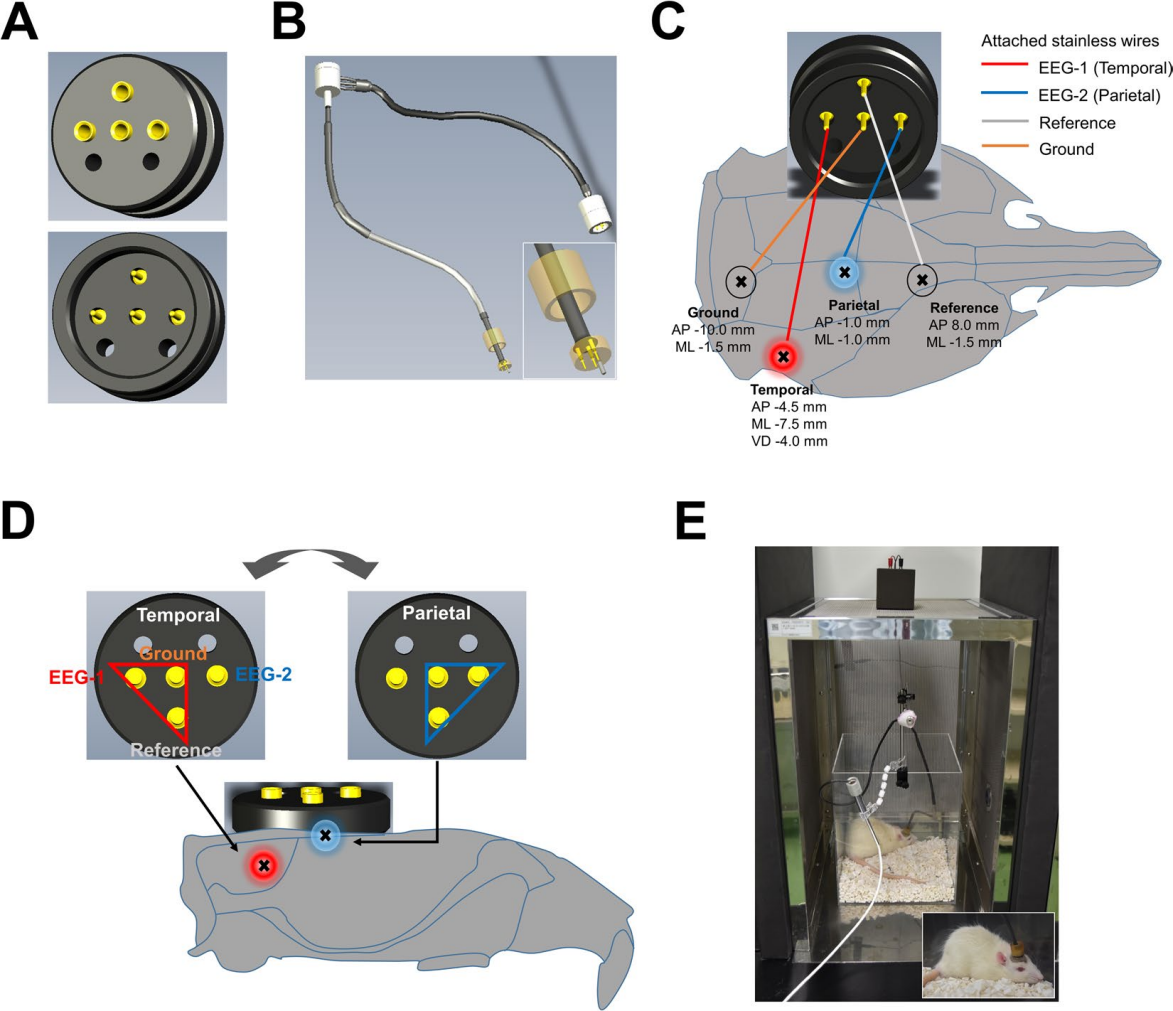
The switchable pedestal which can record from temporal or parietal cortex in the same rat. (**A**) The original switchable pedestal with 4 terminals. (**B**) The customized electrode cables to connect with pedestal. White box shows enlarged illustration of connector pin which attaches to each terminal in the pedestal. (**C**) The anchor screw was put into the skull of rats at the following coordinates: temporal cortex (AP −4.5 mm, ML −7.5 mm, VD −4.0 mm), parietal cortex (AP −1.0 mm, ML −1.0 mm), frontal sinus for reference (AP 8.0 mm, ML −1.5 mm) and cerebellum for ground (AP −10.0 mm, ML −1.5 mm). The stainless steel wires were attached to the pedestal by soldering at the following position: temporal cortex (red line), parietal cortex (blue line), ground (orange line) and reference (gray line) displayed on the figure. (**D**) The recording position of ASSR was switchable between temporal and parietal cortex. On the temporal cortex, customized electrode cables were hooked up with EEG-1, ground and reference (red triangle). On the parietal cortex, customized electrode cables were hooked up with EEG-2, ground and reference (blue triangle). (**E**) Rats were individually placed into a recording box in faraday cage with speakers attached to the cage top, and freely moving during the EEG recording. White box shows enlarged figure of rat with attached electrodes during ASSR recording.

The source of human ASSR has been extensively studied and found to originate in the primary auditory cortex based upon the dipole model^5–9^, the LORETA model^7,9^, and independent component analysis^10^. However, potent ASSR signal has been reported at the vertex (Cz) and/or midline frontal areas (Fz)^8,11–16^. The difference between the highly-responsive brain regions and the generation source region for ASSR are easily reconciled due to the fact that electrocorticogram (ECoG) signal is based on volume conduction^17^. In rodents, the evidence supporting auditory cortex as the ASSR origin region is based on direct implanted electrode recording from multiple brain areas^18^. However, the stable and strict recording of rodent ASSR in ECoG is less well defined considering reports of ASSR from various brain regions such as the prefrontal cortex^19^, and auditory cortex^20,21^. We hypothesized that more precise region-specific analysis would reveal specific electrophysiological phenotypes that could be applied as non-invasive biomarkers for translational studies. Our analysis focused on the parietal and temporal cortex based upon anatomical features of the rat cranium and theory of volume conduction. The application of a novel switchable pedestal enabled recording from these two brain regions from the same rat and consequently resulted in direct power comparison by region (Fig. 1).

Classical auditory ERP analysis produces a signature pattern of time-dependent positive (P1, etc) and negative (N1, etc.) deflections from baseline. The latency or amplitude of these signature peak events can be robustly modulated with tool compounds such as the widely-studied NMDAR antagonist ketamine, that can reduce ERP such as N1 amplitude in both clinical and preclinical studies^22,23^. Through antagonizing NMDAR in the prefrontal cortex, persistent firing of pyramidal neurons can be reduced, affecting neuronal synchrony and disrupting ERP^24^. However, ketamine induced a biphasic effect on inter-trial coherence (ITC) of 40 Hz ASSR in the prefrontal cortex based on the pharmacokinetic/pharmacodynamic (PK/PD) response^19^. Note that the efficacy of ketamine on event-related spectral perturbation (ERSP) in rat ASSR has not been previously reported. The synchronies of ASSR might be influenced by not only the gamma oscillation but also the ERPs. Furthermore, NMDAR antagonism showed the frequency-specific enhancement of ITC in ASSR with four different stimulation frequencies^25^. The current study utilized a wide range frequency stimulus of ASSR to evaluate neural synchronization^26^. ASSR with various different stimulation has been used successfully to drive and examine neural activity across a wide frequency range in healthy volunteers^26,27^, and in case-control studies for schizophrenia^4,28^. Further, these ASSR are sensitive to pharmacological manipulation^29^. Therefore, we also assessed the effect of ketamine over a broad range of ASSR (ERSP and ITC) frequencies in two brain areas using a switchable pedestal to establish this method as a bridge between rats and humans and thus test its utility as a tool for translational and reverse-translational approach between preclinical and clinical studies.

## Results

### Comparison between parietal and temporal cortex recordings of ASSR

**Figure 2:**
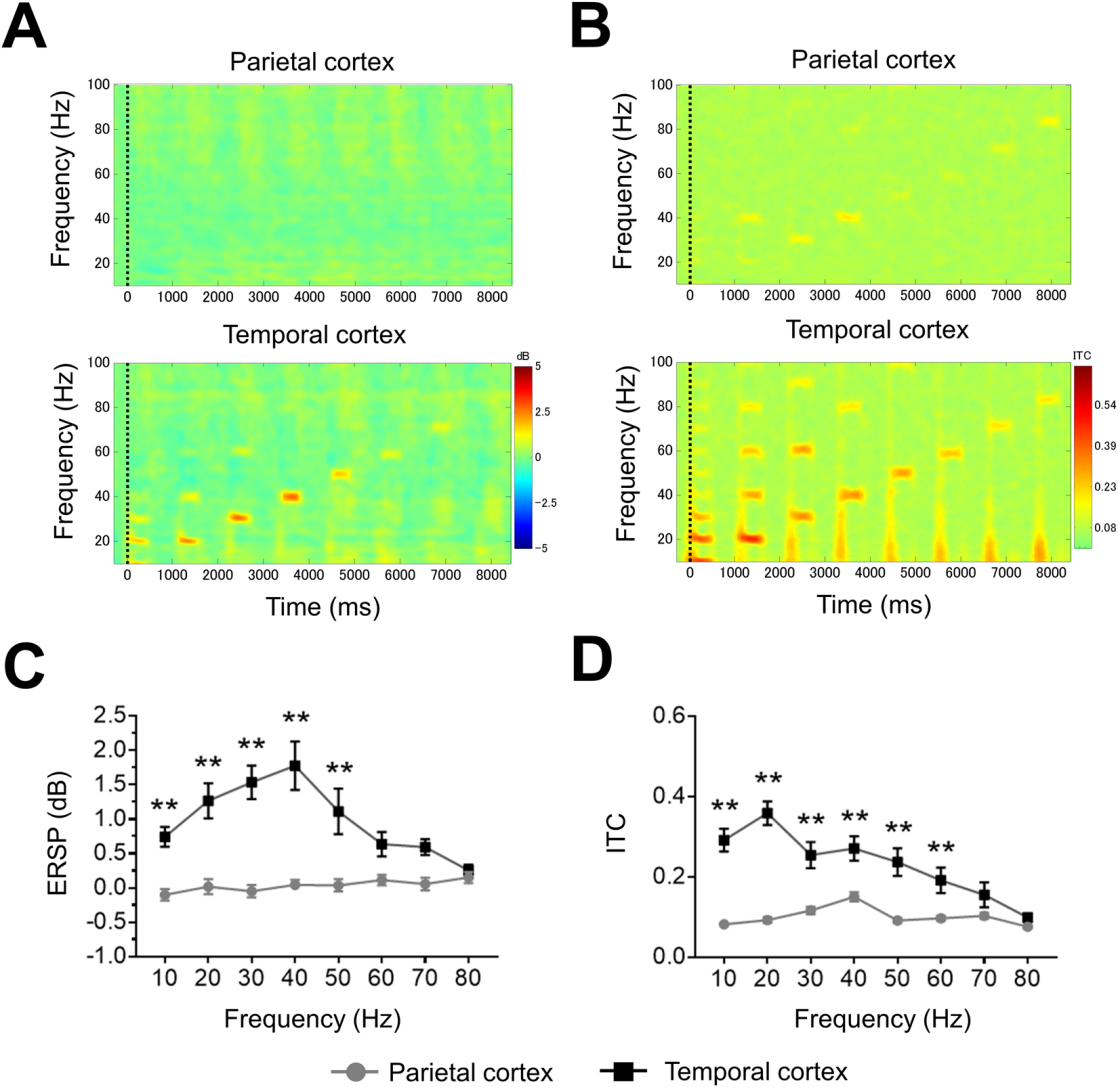
The comparison of ASSR recording between parietal and temporal cortex. (**A**) The time-frequency plots of ERSP at parietal (top) and temporal cortex (bottom). (**B**) The time-frequency plots of ITC at parietal (top) and temporal cortex (bottom). In time-frequency plots, warmer color (reds, yellows) indicate higher ERSP or ITC. (**C**) The ERSP (10–80 Hz) at parietal (circle line) and temporal cortex (square line). (**D**) The ITC (10–80 Hz) at parietal (circle line) and temporal cortex (square line). Data represent mean ± SEM (n = 16). **p < 0.01, significant differences between the groups; two-way repeated measures ANOVA followed by Bonferroni multiple comparison test.

In time-frequency plots, the ASSR from the temporal cortex showed higher ERSP and ITC, whereas neither ERSP nor ITC from the parietal cortex recording showed a clear response (Fig. 2A,B). ASSR between parietal and temporal cortex showed a statistically significant difference by a two-way repeated measures ANOVA with frequency (ERSP; F (7, 105) = 6.632; P < 0.01, ITC; F (7, 105) = 14.34; P < 0.01), and by electrode position (ERSP; F (1, 15) = 33.83; P < 0.01, ITC; F (1, 15) = 41.75; P < 0.01). ASSR from temporal cortex showed significantly higher responses than the parietal cortex in both ERSP (10–50 Hz) and ITC (10–60 Hz). In the gamma band frequency, 40 Hz ASSR showed the most robust response in both ERSP and ITC, although ITC in 20 Hz ASSR showed the maximal response of all frequencies measured (Fig. 2C,D).

### Biphasic alteration of ERSP or ITC in ASSR from temporal cortex recording by ketamine dosing

**Figure 3:**
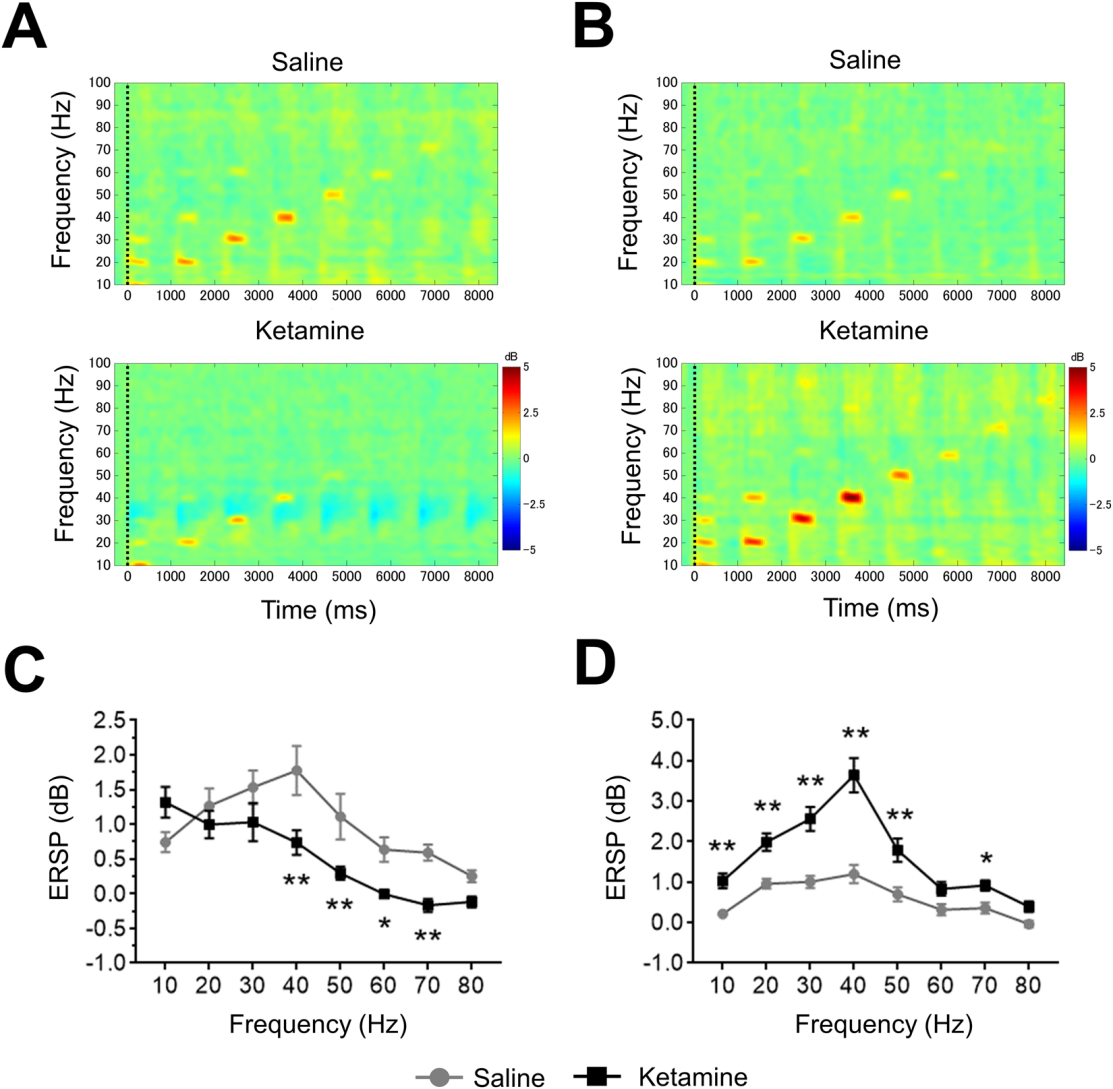
The effect of ketamine on ERSP from temporal cortex recording. (**A**) The time-frequency plots of ERSP at 1^st^ recording (0–30 min) after saline (top) and ketamine (bottom) administration. (**B**) The time-frequency plots of ERSP at 2^nd^ recording (70–100 min) after saline (top) and ketamine (bottom) administration. In time-frequency plots, warmer color (reds, yellows) indicate higher ERSP. (**C**) The ERSP (10–80 Hz) at 1^st^ recording after saline (circle line) and ketamine (square line) administration. (**D**) The ERSP (10–80 Hz) at 2^nd^ recording after saline (circle line) and ketamine (square line) administration. Data represent mean ± SEM (n = 16). *p < 0.05, **p < 0.01, significant differences between the groups; two-way repeated measures ANOVA followed by Bonferroni multiple comparison test.

**Figure 4:**
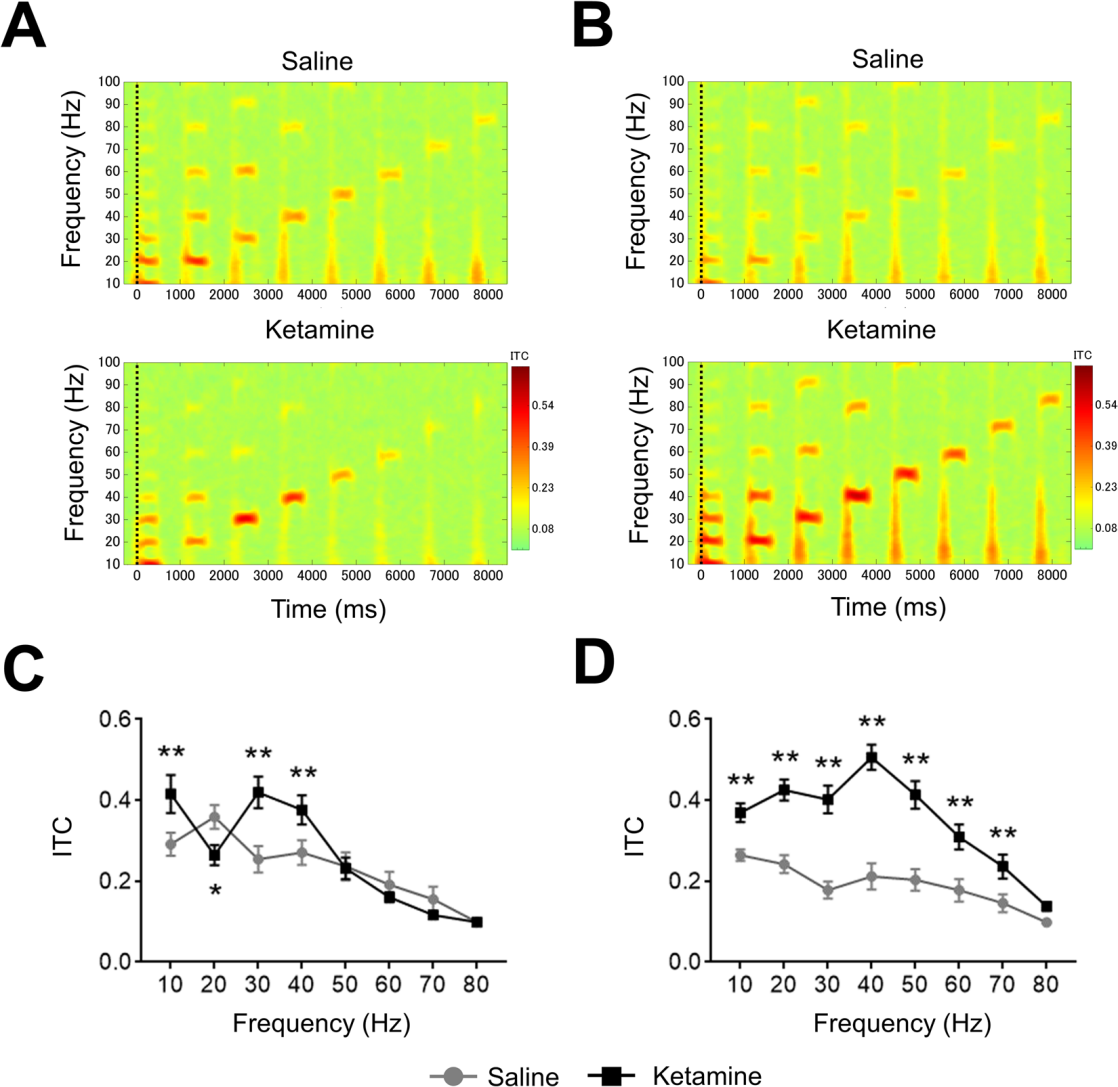
The effect of ketamine on ITC from temporal cortex recording. (**A**) The time-frequency plots of ITC at 1^st^ recording (0–30 min) after saline (top) and ketamine (bottom) administration. (**B**) The time-frequency plots of ITC at 2^nd^ recording (70–100 min) after saline (top) and ketamine (bottom) administration. In time-frequency plots, warmer color (reds, yellows) indicate higher ITC. (**C**) The ITC (10–80 Hz) at 1^st^ recording after saline (circle line) and ketamine (square line) administration. (**D**) The ITC (10–80 Hz) at 2^nd^ recording after saline (circle line) and ketamine (square line) administration. Data represent mean ± SEM (n = 16). *p < 0.05, **p < 0.01, significant differences between the groups; two-way repeated measures ANOVA followed by Bonferroni multiple comparison test.

The alteration of ERSP and ITC by ketamine were recorded at 1^st^ recording (0–30 min) or 2^nd^ recording (70–100 min) after administration at time t = 0 min. In ERSP recording, results were derived from multiple comparison after two-way repeated measures ANOVA with frequency (1^st^ recording; F (7, 105) = 15.28; P < 0.01, 2^nd^ recording; F (7, 105) = 29.51; P < 0.01), treatment (1^st^ recording; F (1, 15) = 7.806; P < 0.05, 2^nd^ recording; F (1, 15) = 55.88; P < 0.01). In ITC recordings, results were derived from multiple comparison after two-way repeated measures ANOVA with frequency (1^st^ recording; F (7, 105) = 30.85; P < 0.01, 2^nd^ recording; F (7, 105) = 23.75; P < 0.01), treatment (1^st^ recording; F (1, 15) = 2.012; P = 0.1765, 2^nd^ recording; F (1, 15) = 80.56; P < 0.01), and frequency × treatment (1^st^ recording; F (7, 105) = 9.095; P < 0.01, 2^nd^ recording; F (7, 105) = 28.84; P < 0.01). Ketamine (30 mg/kg) altered ERSP and ITC in both recording (Figs 3A,B, 4A,B). In the 1^st^ recording, ketamine significantly reduced ERSP at 40, 50, 60, and 70 Hz compared with saline, used here as a solvent control for ketamine (Fig. 3C). ERSP at 10, 20, 30, 40, 50, and 70 Hz significantly increased at 2^nd^ recording after ketamine treatment (Fig. 3D). On the other hand, ITC significantly increased at 10, 30, and 40 Hz in 1^st^ recording by ketamine treatment although ITC at 20 Hz was significantly reduced (Fig. 4C). In the 2^nd^ recording, ketamine significantly increased ITC at 10, 20, 30, 40, 50, 60 and 70 Hz compared with saline (Fig. 4D).

### The alteration of ASSR from parietal cortex recording by ketamine administration

**Figure 5:**
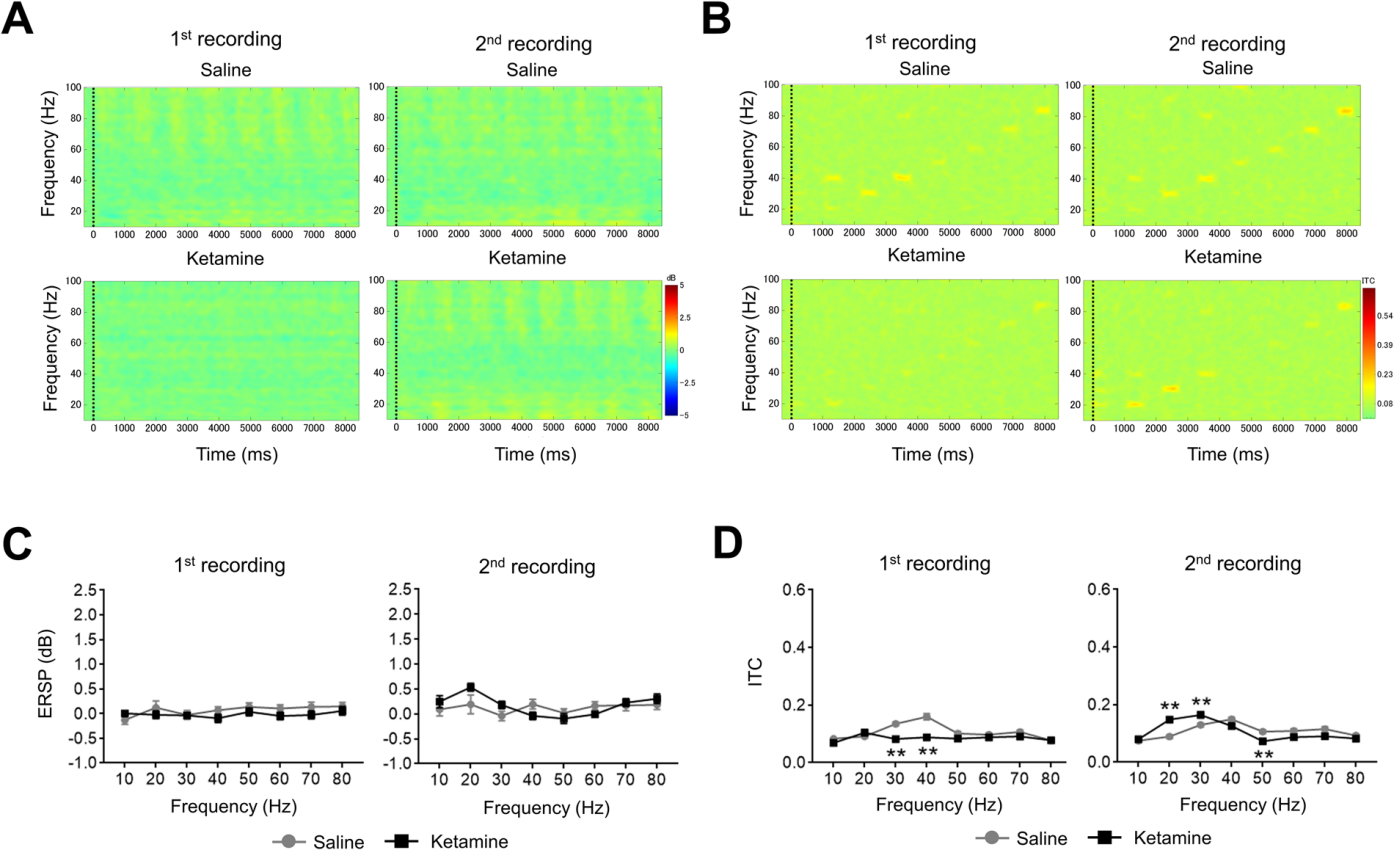
The effect of ketamine on ASSR from parietal cortex. (**A**) The time-frequency plots of ERSP at 1^st^ recording (0–30 min) and 2^nd^ recording (70–100 min) after saline (top) and ketamine (bottom) administration. (**B**) The time-frequency plots of ITC at 1^st^ recording (0–30 min) and 2^nd^ recording (70–100 min) after saline (top) and ketamine (bottom) administration. In time-frequency plots, warmer color (reds, yellows) indicate higher ERSP and ITC. (**C**) The ERSP (10–80 Hz) at 1^st^ recording and 2^nd^ recording after saline (circle line) and ketamine (square line) administration. (**D**) The ITC (10–80 Hz) at 1^st^ recording and 2^nd^ recording after saline (circle line) and ketamine (square line) administration. Data represent mean ± SEM (n = 16). **p < 0.01, significant differences between the groups; two-way repeated measures ANOVA followed by Bonferroni multiple comparison test.

**Figure 6:**
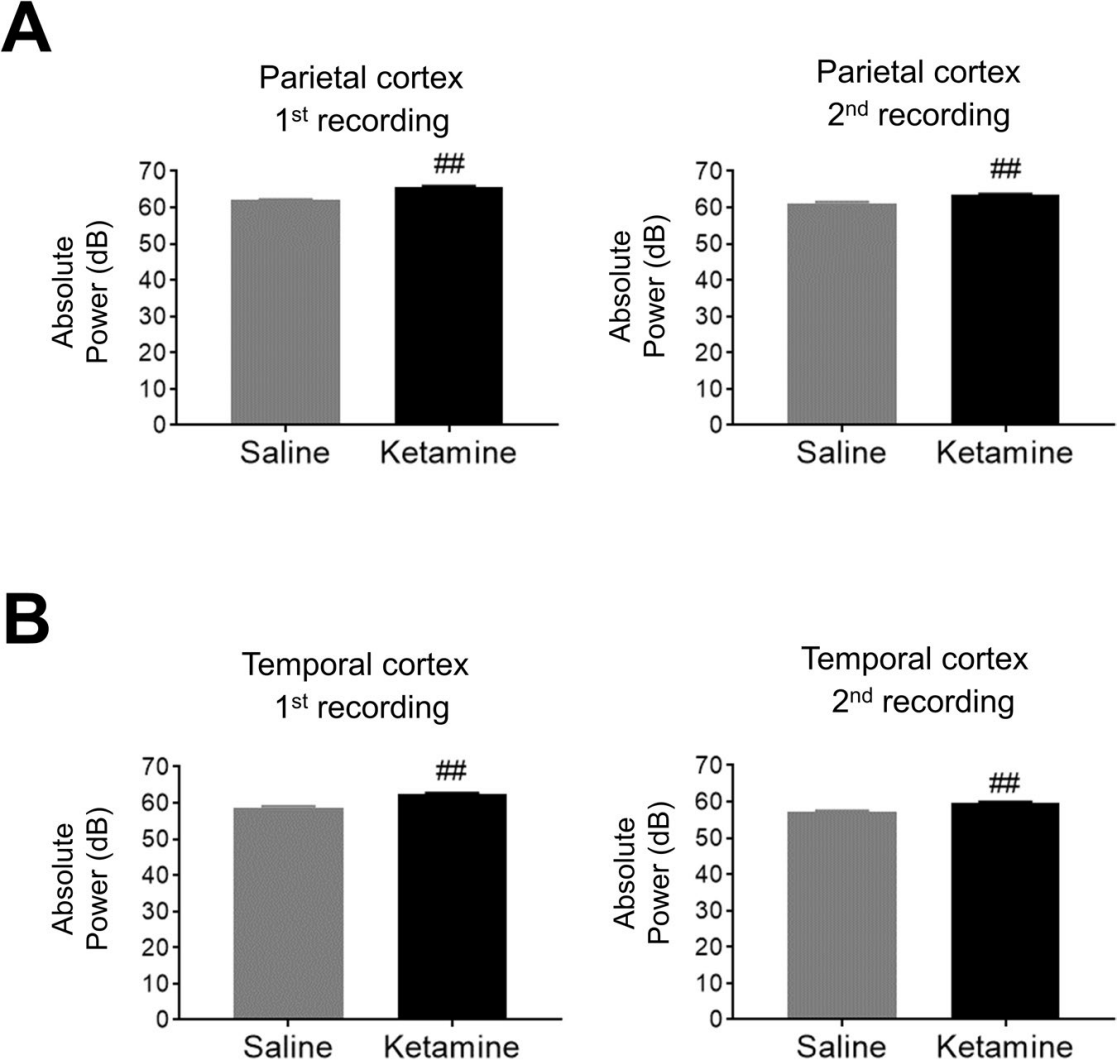
The effect of ketamine on basal power of gamma frequency bands (30–80 Hz). (**A**) The absolute low gamma power (30–80 Hz) at 1^st^ recording or 2^nd^ recording after saline (left) or ketamine administration (right) in parietal cortex. (**B**) The absolute low gamma power at 1^st^ recording or 2^nd^ recording after saline (left) or ketamine administration (right) in temporal cortex. Data represent mean ± SEM (n = 15–16). ^##^p < 0.01, significant differences between the groups; paired t-test.

Ketamine did not alter ERSP in both recording of ASSR recording from parietal cortex after two-way repeated measures ANOVA with frequency (1^st^ recording; F (7, 105) = 0.8242; P = 0.57, 2^nd^ recording; F (7, 105) = 3.136; P < 0.01), treatment (1^st^ recording; F (1, 15) = 5.203; P < 0.05, 2^nd^ recording; F (1, 15) = 0.405; P = 0.53) (Fig. 5A,C). In the ITC recording, results were derived from multiple comparison after two-way repeated measures ANOVA with frequency (1^st^ recording; (F (7, 105) = 10.8; P < 0.01, 2^nd^ recording; F (7, 105) = 26.91; P < 0.01), treatment (1^st^ recording; F (1, 15) = 52.92; P < 0.01, 2^nd^ recording; F (1, 15) = 0.205; P = 0.66), and frequency × treatment (1^st^ recording; F (7, 105) = 9.986; P < 0.01, 2^nd^ recording; F (7, 105) = 11.8; P < 0.01). ITC during 1^st^ recording showed significant reduction at 30 and 40 Hz (Fig. 5B,D). These reductions of ITC remained in the 2^nd^ recording with significant reduction at 50 Hz although ITC at 20 and 30 Hz was significantly increased by ketamine treatment. However, the basal power of gamma frequency bands (30–80 Hz) was significantly increased at the temporal cortex as well as parietal cortex in both recordings after ketamine administration (Fig. 6A,B).

## Discussion

The innovative feature of our study is that our switchable pedestal enabled recording from both parietal and temporal cortex in the same rat without restraint. This enabled us to probe suitable recording position for rat ASSR and establish the potential for application of ASSR as a translational biomarker. The robust response from rat temporal cortex in the 40 Hz range is consistent with ASSR in human^26^. From our wide-range ASSR paradigm, both shared and unique features between preclinical and clinical assessments were discovered. The temporal cortex recording of ASSR produced a biphasic effect of ketamine on ERSP/ITC while the parietal recording from the same animals did not.

### Temporal cortex recording in ECoG enables neuronal synchronization of ERSP and ITC

ASSR recording from the temporal cortex revealed significantly higher ERSP and ITC at frequency of 10–50 Hz compared to values recorded from the parietal cortex. Supporting this, Wang *et al*. suggested that ASSR recording from the auditory cortex showed the highest response by comparison with other brain regions such as prefrontal cortex and hippocampus, among others^18^. Prior studies reported clear ERSP in rat ASSR^21,25^. However, our ECoG recording methods significantly contribute to this literature by enabling detection of both ERSP and ITC from temporal cortex. In contrast, the parietal recording of ASSR produced unclear ERSP and ITC. We first clarified the different responses of ERSP and ITC in these regions in rodent. However, potent response of ASSR were detected at the vertex and/or midline frontal areas in clinical studies^8,11–16^. The difference of the high-response region might be derived from shape and/or volume differences of the brain between rats and human because volume conduction effects enable ECoG recording^17^. Indeed, threshold differences in bone-conduction ASSR between post-term infants and adults has been suggested to be due to skull maturation^8,30^. By using the switchable pedestal, we revealed the detectable signal from rat ASSR in temporal cortex, and difference of ASSR between rats and human. These results shown here emphasize the utility of present methodology for the basic science of ASSR.

### Frequency characteristics of ASSR in preclinical and clinical investigations

The wide-range frequency of ASSR setup enables clarification of common and divergent features between preclinical and clinical studies. Common features include the peak ERSP and ITC responses at 40 Hz in the gamma band in rat and human ASSR^26,28^. Divergent features include the highest ITC at 20 Hz and smallest ERSP and ITC at 80 Hz in rat which were not consist with the clinical reports although the same results were reported in rat ASSR^25^. These shared and unique features indicate that ASSR can be a powerful translatable biomarker, but that care must be taken to select the most informative frequencies for stimulation and response.

### Alternation of ASSR over broad frequencies by ketamine administration

Ketamine at 30 mg/kg in rat significantly decreased the ERSP at 40–70 Hz in the 1^st^ recording. However, the response to ketamine was inverted significantly in the 2^nd^ recording. We first revealed the biphasic effect at gamma bands in ASSR from temporal cortex recording. This result is consistent with a previous report that observed biphasic responses in 40 Hz ASSR despite recording from Fz^19^. Ketamine altered both amplitude and latency in auditory ERPs as a shared feature between clinical and preclinical studies^22,23^. This effect of ketamine might be caused by the persistent reduction of neuronal firing of inter neurons, i.e. GABAergic neurons, in the cortex^1,24,31^. Thus, ketamine might directly influence the mode of action by which gamma oscillation is developed in ASSR. It has been suggested that lower exposure of ketamine (like 2^nd^ recording in present study) biases the excitatory/inhibitory balance toward increased excitability^19^. However, the effect of ketamine on ASSR could not be only explained by the inhibition of neuronal firing, because it produced biphasic responses across a wide range of gamma band frequency. Indeed, higher exposure of ketamine (1^st^ recording) reduced both ERSP and ITC of ASSR. This may reflect a collapse of cortical neuronal synchrony. However, it is necessary to investigate this result at a deeper mechanistic level to better understand the dose- and time-dependent differential responses as they could simply be caused by a specific efficacy of ketamine such as dissociative anesthesia.

Studies of gamma frequency oscillation (40 Hz) have demonstrated differences between schizophrenic patients and normal controls^32–34^. One conclusion from these studies is that inappropriate regulation of fast-spiking cortical GABAergic interneurons results from NMDA hypofunction. The present study showed reduction of the 40 Hz ERSP consistent with schizophrenia patients and robustly affected the ERSP and ITC at 30 and 40 Hz by ketamine. In addition, augmentation of ASSR at 40 Hz in the 2^nd^ recording was also consistent with clinical observations^35^. These results indicate that our recording methods is able to detect high similarity of ASSR in schizophrenia patients and robust alteration of gamma oscillations.

This methodology also revealed that the augmented ERSP and ITC at 10 Hz ASSR by ketamine remained in both recordings. To the best of our knowledge, there are no previous reports demonstrating that ketamine affects 10 Hz ASSR in both preclinical and clinical experimental systems. Ketamine altered ERPs in both clinical and preclinical studies^22,23^ and basal power of alpha bands (8–12 Hz) in clinical studies^36^. This could reflect a causal relationship among ERPs, basal power and ASSR, but further investigation is warranted.

ASSR recording from parietal cortex did not show a robust biphasic effect of ketamine on ERSP and ITC. However, the basal power in the gamma frequency was significantly increased in parietal cortex recording as well as temporal cortex recording by ketamine in the same rat. A previous report also suggested that 30 mg/kg ketamine steadily augmented gamma power in frontal cortex^37^, suggesting that the exposure level of ketamine in our study is suitable for producing neurophysiological responses. Thus, the switchable pedestal with a wide range of frequencies of sound stimuli revealed that the rat ASSR has not only regional differences but also differences of drug responses. Through direct intracranial comparison of candidate brain regions, sampling a broad range of input stimuli frequencies and output response frequencies, the current study identified the temporal (preclinical) and parietal/Fz (clinical) as a suitable brain regions and 40 Hz a tractable frequency for translational studies linking informative features of rat electrophysiological response to standard noninvasive measurements of human clinical response to drug. Further investigation by using the novel device described here to test hypotheses in additional brain areas such as the prefrontal cortex will provide insights into the mechanism of ASSR, and its interpretation/validation as a translatable biomarker.

The critical need for more predictive human biomarkers of neuroleptic drug efficacy is matched by a need for preclinical experimental systems that accurately reflect the human condition. Multiple consortia of academic research teams and federal programs are driving toward the application of human EEG/ERP as clinical biomarkers. These include the Bipolar Schizophrenia Network on Intermediate Phenotypes (BSNIP) project @ NIMH, ERP Biomarker Qualification Consortium, Brain/MINDS project^38–40^. Outside of the basic research domain and in the context of neuroleptic drug development, there will be a growing need for corresponding preclinical experimental systems that provide a clear line of sight from drug discovery through development. This work takes an important step toward harmonizing ASSR as a translational tool toward achieving this ambitious end.

## Methods

### Animals

Sprague-Dawley rats were purchased (Charles River Laboratories Japan, Inc., Ishioka, Japan). The sixteen rats were housed in temperature- and humidity-controlled rooms (23 ± 2 °C and 55 ± 10%) under a 12-h light/dark cycle with free access to water. Recording were performed in dim light (<300 lux). All animal experimental procedures were performed in accordance with Guide for the Care and Use of Laboratory Animals, 8th edition and approved by the Institutional Animal Care and Use Committee of Astellas Pharma Inc. Furthermore, Astellas Pharma Inc., Tsukuba Research Center was awarded Accreditation Status by the AAALAC International.

### Surgery

We created an original electrode pedestal which can switch the acquisition position between temporal cortex or parietal cortex (Fig. 1A) and customized electrode cables (Fig. 1B) in cooperation with S.E.R. Corporation (Shinagawa-ku, Tokyo, Japan). Design details can be made available upon request. Before 8 weeks of age, rats underwent anchor screw attachment into the skull (no contact with the meninges) at the following coordinates: temporal cortex (AP −4.5 mm, ML −7.5 mm, VD −4.0 mm), vertex (AP −1.0 mm, ML −1.0 mm, parietal cortex), frontal sinus for reference (AP 8.0 mm, ML −1.5 mm) and cerebellum for ground (AP −10.0 mm, ML −1.5 mm) under anesthesia using 2–2.5% isoflurane (Fig. 1C). The stainless steel wires were attached to the switchable pedestal by soldering at each terminals (Fig. 1C). The switchable pedestal with stainless steel wire electrodes were attached to the cranium using methacrylic resin (Repairsin®, GC Corporation, Bunkyo-ku, Tokyo, Japan). After surgery, the rats were housed singly and had unrestricted access to food and water.

### Drugs

Ketamine hydrochloride (50 mg/mL, Daiichi Sankyo Co., Ltd, Tokyo, Japan) was diluted with saline. Ketamine (30 mg/2 mL/kg) or saline were subcutaneously administered to all rats in a final volume of 2 ml/1 kg body weight.

### EEG recording

EEG recording was performed using a programming script with a data acquisition and analysis software package (Spike2®, Cambridge Electronic Design, Milton, Cambridge, UK). Rats were hooked to customized electrode cables up to the switchable pedestal, selecting the temporal cortex or parietal cortex with ground and reference (Fig. 1D). The electrode cables were connected to high-impedance differential AC amplifier (Sampling rate; 1000 Hz, Low Cut-Off Filter: 1 Hz, High Cut-Off Filter: 500 Hz, model #1800, A-M Systems, Carlsbrog, WA, US) and versatile data acquisition unit (Micro1401, Cambridge Electronic Design). Rats were individually placed into recording boxes in a faraday cage with speakers attached to the cage top, and freely moved during the EEG recording (Fig. 1E). For habituation, the ASSR recording was started at least 30 min after placement in the recording box. Auditory stimuli consisted of click sounds (80 dB) which include 500 msec trains at 10, 20, 30, 40, 50, 60, 70, and 80 Hz. Click sounds trains at each frequency were repeated 200 times/trial, the inter-trial interval (ITI) and inter-signal interval (ISI) was 600 msec. Rats were exposed to auditory sound stimuli at 0–30 (1^st^ recording) and 70–100 min (2^nd^ recording) automatically by the programming script after saline administration, and the EEG data were recorded from parietal or temporal cortex. After that consecutively, rats were treated with ketamine and were re-exposed to the auditory sound stimuli. At least one week without recording was applied to all rats as a withdrawal term between recordings from parietal or temporal cortex.

### Data analysis

The EEG data from Spike2 were converted to a Matlab environment. By using EEGLAB® on a MATLAB toolbox (MathWorks, Natick Massachusetts, USA), the files were split into each trial (200 times), all outliers in each split file were rejected for movement artifact based on a criterion of 2 times root mean squared amplitude per mouse. In ASSR analysis, low-pass filter (100 Hz) was applied to the EEG data to remove artifacts. The mean number of epochs for each group were as follows: parietal (saline at 1^st^ recording: 181, ketamine at 1^st^ recording: 186, ketamine at 2^nd^ recording: 180), temporal (saline at 1^st^ recording: 183, ketamine at 1^st^ recording: 188, ketamine at 2^nd^ recording: 179). Averaging stimuli during each click sound trains were calculated by wavelet transformation (frequency limits: 8 to 100 Hz, wavelet cycles:10 to 62.5, epoch size: 9.0 sec). As output data, the measurable factors were divided into two different data; event-related spectral perturbation (ERSP) (baseline: pre-stimulations from −600 to 0 msec) and inter-trial coherence (ITC) on a MATLAB toolbox and were gathered by open source software, KNIME® (KNIME AG, Zurich, Switzerland). By using same split files, spectrum analysis was performed and calculated by FFT analysis (baseline: pre-stimulations) on MATLAB toolbox. The value during 30–80 Hz were derived from power of spectrum as basal gamma power.

### Statistics

Statistical analysis for ASSR between the parietal cortex group and the temporal cortex group, or saline group and ketamine group, was conducted using two-way repeated measures ANOVA followed by Bonferroni multiple comparison test (GraphPad Prism 7®, GraphPad Software, San Diego CA, USA). Statistical analysis for basal gamma power between saline group and ketamine group was conducted using paired t-test.

## Conclusions

We constructed and validated novel methods for ASSR recording by using a switchable pedestal which allows ASSR recording at the temporal cortex or parietal cortex with a wide range of frequencies in same freely moving rat. As the results, ASSR recording at the temporal cortex enabled distinct detection of ERSP and ITC with each auditory click train stimuli, and that the 40 Hz ASSR shows maximal ERSP and ITC, similar to humans. Ketamine exerted a bi-phasic effect in gamma bands (40–70 Hz). These responses and effects were not clearly detected in ASSR recordings from parietal cortex in the same rats. Thus, ASSR recordings from the temporal cortex have high validity as a preclinical ASSR model. From these findings, our constructed switchable recording and wide-range ASSR paradigm are useful for basic science and novel drug development as a forward and backward translational science tool.
